# Variations of freezing tolerance and sugar concentrations of grape buds in response to foliar application of abscisic acid

**DOI:** 10.3389/fpls.2023.1084590

**Published:** 2023-02-17

**Authors:** Imed Dami, Yi Zhang

**Affiliations:** ^1^ Department of Horticulture and Crop Science, The Ohio State University, Wooster, OH, United States; ^2^ Grapery, Shafter, CA, United States

**Keywords:** cold hardiness, Chambourcin, Cabernet franc, raffinose, sugars, ABA

## Abstract

The purpose of this study was to explore the mechanism of ABA-induced freezing tolerance increase in grapevines. The specific objectives were to evaluate the impact of ABA treatment on soluble sugar concentration in grape buds and determine the correlations between freezing tolerance and ABA-affected soluble sugar concentration. *Vitis spp* ‘Chambourcin’ and *Vitis vinifera* ‘Cabernet franc’ were treated with 400 and 600 mg/L ABA in the greenhouse and field. The freezing tolerance and soluble sugar concentration of grape buds were measured monthly during the dormant season in the field and at 2wk, 4wk, and 6wk after ABA application in the greenhouse. It was observed that fructose, glucose, and sucrose are the main soluble sugars that correlate with freezing tolerance of grape buds and the synthesis of these sugars can be enhanced by ABA treatment. This study also found that ABA application can promote raffinose accumulation, however, this sugar may play a more important role in the early acclimation stage. The preliminary results suggest that raffinose accumulated first in buds, then its decrease in mid-winter corresponded with the increase of smaller sugars, such as sucrose, fructose, and glucose, which in turn, corresponded with reaching maximum freezing tolerance. It is concluded that ABA is a cultural practice tool that can be used to enhance freezing tolerance of grapevines.

## Introduction

Cold damage is by far the most devastating weather event to grape production (Snyder and de Melo-Abreu, 2005). Grapes and the established wine reputation of certain regions are sensitive to climate extremes, and there are concerns about how changing climate patterns will impact these industries. Although average winter temperatures have been trending upwards over the last 20 years, so has the variability in winter temperatures (https://mrcc.purdue.edu). The rise of average temperature has a two-fold impact on grapevines: it retards cold acclimation in the fall and reduces winter freezing tolerance (FT), resulting in a faster deacclimation and earlier budburst, which renders grapevines more vulnerable to spring frost (Wolf and Cook, 1992; Kovaleski et al., 2018).

To mitigate freezing stress, grapevines adapt to cold climates by undergoing physiological changes that result in a transition from a cold-sensitive to a cold-hardy state, a process known as *cold acclimation*. During this process, nearly 800 genes are upregulated and 2300 genes are downregulated (Londo et al., 2018). During cold acclimation, plants increase their desiccation and freezing tolerances by involving various physiological and biochemical changes, such as sugar accumulation. A decrease in photoperiod (*i.e.* daylength) has been reported to induce the synthesis of the phytohormone ABA, which is then translocated to the various plant tissues, resulting in shifts in gene expression and a cascade of cellular signaling responses (Chao et al., 2007). ABA has been suggested to play a role during these changes by promoting soluble sugar accumulation (Xue-Xuan et al., 2010). Soluble sugars, including sucrose, glucose, fructose, and raffinose family oligosaccharide (RFO) such as raffinose and stachyose accumulate when plants develop freezing tolerance in winter and decrease during deacclimation in spring (Wanner and Junttila, 1999). Among these soluble sugars, sucrose and raffinose have been suggested to play an important role in cold acclimation of woody plants. A sudden increase of sucrose and raffinose concentrations at the start of cold acclimation was observed in poplar wood (*Populus x canadensis Moench* ‘‘robusta’’) and remained high during the winter season (Sauter et al., 1996). Furthermore, cold tolerant grapevines were found to accumulate more soluble sugars and have differential expression of sugar metabolism genes compared to cold-sensitive plants (Chai et al., 2019). Genes involved in sugar metabolism, such as galactinol synthase (GolS), raffinose synthase (RafS), β-amylase (BAMY), and phosphoglycerate kinase (PGK), were differentially expressed under freezing temperatures (Londo et al., 2018; Chai et al., 2019; Wang et al., 2021).

The functional role of soluble sugars during cold acclimation has been proposed as osmotic regulator and cryoprotectant. It has been reported that soluble sugars can be used to adjust osmotic pressure in leaf and root cells under water stress (Ogawa and Yamauchi, 2006). It has also been suggested that some soluble sugars, such as sucrose, can interact with the lipid bilayer of cell membranes to prevent damage caused by dehydration (Anchordoguy et al., 1987). Furthermore, the hydrogen bond between glucose and protein can stabilize protein structure and prevent dehydration-induced protein unfolding (Allison et al., 1999). As cryoprotectants, sugars can prevent ice crystallization by inhibiting the nucleation of ice crystals. In this case, the water in cells can be solidified as an amorphous glass (Sacha and Nail, 2009).

Endogenous ABA has been suggested to play an important role in promoting the production of soluble sugars during cold acclimation of many plant species, such as Lily (*Lilium rubellum* L.) and moss (*Physcomitrella patens* L.) (Xu et al., 2006; Bhyan et al., 2012). It has also been reported that exogenous ABA application (40 and 100 mg/L) induced the accumulation of fructose and sucrose in wheat (*Triticum aestivum* L.) along with increased freezing tolerance (Kerepesi et al., 2004). In grape (*Vitis vinifera*), sucrose and raffinose have been found to correlate with the variation of the ABA content in buds and ambient temperature during dormant season (Koussa et al., 1998). In gentian (*Gentiana scabra* L.), it has also been found that the concentrations of sucrose and raffinose in buds are sensitive to ABA application since incubating buds with ABA solution increases the sucrose and raffinose concentration with increased desiccation tolerance (Suzuki et al., 2006). Moreover, applying ABA inhibitor (fluridone) decreased the sugar concentration and desiccation tolerance (Suzuki et al., 2006).

Additionally, the ABA-inducible raffinose production in seed embryos in relation to desiccation tolerance is well documented. The seedlings grown from exogenous ABA-incubated cucumber (*Cucumis sativus* L.) seeds showed higher raffinose concentration in tissues with a higher desiccation tolerance than control groups (Wang et al., 2012). There is evidence showing that the ABA-activated galactinol synthase is mainly responsible for the accumulation of raffinose in seeds (Blochl et al., 2005).

Previous greenhouse and field studies demonstrated that exogenous ABA application advanced cold acclimation and increased freezing tolerance of grapevines (Zhang et al., 2011; Zhang and Dami, 2012a). In this study, the relationship between ABA and sugar metabolism was investigated. The specific objectives were to: 1) evaluate the effect of exogenous ABA on soluble sugar concentration in grape buds of ‘Chambourcin’ and ‘Cabernet franc’ cultivars; and 2) determine the correlations between freezing tolerance and ABA-affected soluble sugar concentration.

## Materials and methods

### Plant materials, experimental design, and treatments

This study consisted of two field experiments conducted during the dormant season and one greenhouse experiment.

#### Greenhouse experiment

One-year-old dormant *Vitis vinifera* ‘Cabernet franc’ grafted on *Vitis riparia* × *Vitis rupestris* ‘Couderc 3309’ were planted in 7.6 L pots and placed on benches in the greenhouse. In the second year, one-year-old own-rooted *Vitis spp* ‘Chambourcin’ grapevines were also planted under the same conditions. The greenhouse conditions were consistent with the settings described in Zhang et al. (2011). Briefly, the greenhouse settings were as follows: 22/19°C and 50/50% relative humidity (day/night). The light intensity was maintained at 300 umol.m^2^. s^-1^ using metal halide 1000-W high-pressure sodium lights (Sunlight Supply, Woodland, AZ). All grapevines were pruned back and kept in 4°C cooler to satisfy their chilling requirements then placed in the greenhouse to promote budburst.

The grapevines were pruned to the twelfth basal nodes when the leaf age was approximately 50 days. ABA was applied with the concentration of 0 (control) and 400 mg/L. All the ‘Chambourcin’ and ‘Cabernet franc’ grapevines were sprayed when the leaf age was approximately 80 days. Prior to applying ABA, the average leaf number and shoot length per vine were 18 and 140 cm, respectively. Leaf and bud samples were collected at 2, 4, and 6wk after ABA application, corresponding to leaf age of 94, 108, and 122 days, respectively. At each sample collection time, four vines from control and ABA treated groups were randomly selected. The experimental design was a completely randomized design. Buds on node positions 1 to 5 were used for the freezing tests. Leaves and buds on the same position on the adjacent shoot of the same vine were used for sugar analysis.

Leaf age was determined based on Eichorn-Lorenz (EL) stages of shoot development (Eichorn and Lorenz, 1977) with one-day leaf age corresponding to the first unfolded leaf (EL stage 7) originating from the third basal node.

The same ABA sample and surfactant from the field experiments were used in the greenhouse experiment. Whole vines were sprayed with ABA solutions to run off with a 7.6 L hand-held sprayer (Gilmour Gardening Innovation, Peoria, IL) averaging a spray volume of 0.2 L/vine.

#### Field experiment

During 2010-2011 dormant season, two field experiments were conducted at the Research Vineyard in Wooster and the Ashtabula Agricultural Research Station, Kingsville, OH. In Wooster, OH, grafted ‘Chambourcin’ (Seyve-Villard 12417 × Seibel 7053) grapevines on *Vitis riparia* × *Vitis rupestris* rootstock ‘Couderc 3309’ and planted in 1996 at the Research Vineyard, were used for this study. Vines were spaced 1.25 × 3 m (vine × row), trained to high-cordon system (height = 1.83 m), and spur-pruned to 2 buds per spur and 16 buds per meter of cordon, followed by shoot and cluster thinning to 13 and 20 per meter of cordon, respectively prior to ABA treatment. Seven ABA treatments were assigned to vines on a randomized complete block consisting of four blocks with 5 vines per plot unit as follows: control (deionized water), 400 and 600 mg/L ABA sprayed at 50% véraison stage (4V and 6V, respectively), 400 and 600 mg/L ABA sprayed at 20 days after véraison stage (4V20 and 6V20, respectively), 400 and 600 mg/L ABA sprayed at 40 days after véraison stage (4V40 and 6V40, respectively). In Kingsville, OH, ‘Cabernet franc’ (Clone 1) grafted on *Vitis riparia* × *Vitis rupestris* 101-14 Millardet et de Grasset rootstock were planted in 2005. Vines were spaced 1.8 × 2.4 m (vine × row), trained to a bi-lateral cordon system with vertically-shoot positioned, and spur-pruned to 16 buds/m of cordon. The vineyard block was divided into four blocks. Each block consisted of 20 grapevines, which were divided into five panels (four vines per panel). Each panel was randomly assigned to one of five treatments: control (same as above), 600 mg/L ABA sprayed at V, V20, V40, and V55 (20, 40, and 55 days after 50% véraison stage). In each panel, the first three vines were used as a replicate for one treatment and the fourth was an untreated buffer vine. Canopy management practices consisted of leaf removal of the basal three leaves on both sides of the canopy in late July and shoot hedging performed in early August. A spring frost event (-1.2°C) occurred on 10 May 2010 which caused injury of shoots and inflorescences and resulted in uneven number of clusters per vine. In order to avoid the potential confounding effect of crop level, the shoot number per vine was adjusted and all clusters were removed from all treated vines. Daily temperatures from weather stations at the vineyard sites were recorded during the field experiments (**Supplemental Figures 1**, **2**).

The ABA sample (VBC-30051) was provided by Valent Bioscience (Libertyville, IL). The a.i. was 20.0% (w/w) S-ABA. The ABA sample was dissolved in deionized water with 0.05% Tween-20 (Acros Organic, Hampton, NH). Whole vine canopies (leaves and clusters) were sprayed with ABA solutions to runoff with a 15-L back sprayer (SP System LLC. Model SP0, Santa Monica, CA) averaging a spray volume of 0.5 L/vine.

### Determination of freezing tolerance

In the field experiments, one representative one-year-old cane with a minimum of 12 to 15 lignified internodes was collected from each replication and buds on node positions 3 to 7 were used. There were five buds used from each replication and 20 buds per treatment in both ‘Chambourcin’ and ‘Cabernet franc’. Buds were excised and mounted on thermoelectric modules (MELCOR, Trenton, N.J.), which were placed in a Tenney environmental chamber (Thermal Products Solutions, New Columbia, PA). The chamber temperature was lowered from -2 to -50°C at 4°C/hr. Freezing tolerance of buds was determined using differential thermal analysis and was expressed as the average lethal temperature exotherm that kills 50% of the bud population or LT50 (Wolf and Pool, 1987). For Chambourcin, LT50s were determined five times from October 2010 to February 2011. For Cabernet franc, LT50s were determined six times from October 2010 to January 2011. In the greenhouse experiment, the buds were excised and tested following the same procedures applied in the field experiments.

### Sugar analysis

In the field experiments, the cane selection followed the same protocol applied in freezing tolerance determination. The buds were excised and frozen immediately in a box with dry ice and then stored at -80°C freezer. In the greenhouse experiment, the buds and leaves were immediately plunged in liquid nitrogen and then stored at -80°C freezer. The sugar extraction, derivatization, and quantification followed a modified protocol based on Grant et al. (2009).

#### Extraction

Five frozen buds were ground by mortar and pestle in liquid nitrogen and then freeze dried. The freeze-dried buds were weighed and transferred to a 2-ml microcentrifuge tube before extraction. Leaf samples were freeze dried first and then ground to powder. For each replicate/treatment, approximate 5-6 mg leaf samples were weighed and transferred to a 2-ml microcentrifuge tube before extraction. One mL of 75% ethanol (Fisher Scientific, Pittsburgh, PA) was added to bud and leaf samples and the samples left at room temperature for 3h and shaken every 30 min. The samples were then centrifuged at 6708 x g for 10 min and the supernatants were transferred to a glass vial for collection. The extraction procedure was repeated twice and the glass vials ended up with approximate 2-3 mL supernatant collection. The glass vials were placed on the Reacti-Therm Heating Modules (Thermo Fisher Scientific, Waltham, MA) at 45°C and dried under an air stream overnight.

#### Derivatization

For bud samples, 250 μL pyridine (Sigma-Aldrich, St. Louis, MO) and 250 μL STOX solution (For 750 μg/vial internal standard: 100 mL pyridine, 2.5 g hydroxylamine hydrochloride, and 0.6 g phenyl-β-D-glucopyranoside; For 100 μg/vial internal standard: 100 mL pyridine, 2.5 g hydroxylamine hydrochloride, and 80 mg phenyl-β-D-glucopyranoside) were added to each dried glass vial. The vials were shaken for 10 sec and placed on the Reacti-Therm Heating Modules at 70°C for 40 min. Vials were then removed from the heating block and cooled under room temperature. Four hundred μL hexamethyldisilazane (HMDS, Sigma-Aldrich, St. Louis, MO) and 40 μL trifluoroacetic acid (TFA, Sigma-Aldrich, St. Louis, MO) were added to each glass vial and the vials were shaken for 10 sec. Finally, the vials were placed at 4°C refrigerator overnight for precipitation. The supernatants were transferred to 1.5 mL vials and ready for sugar analysis the next day. Leaf samples were derivatized following the same procedure except the volumes for pyridine, STOX solution, HMDS, and TFA were 125, 125, 200, and 20 μL respectively.

#### Gas chromatography/Flame ionized detector

GC/FID was used to analyze samples from the field and 2011 greenhouse experiments. The derivatives were injected to a gas chromatograph (Hewlett Packard 5890 Series II, Hewlett Packard, Boulder, CO) with a 30-m capillary column (HP5-MS, 250 μm inner diameter and 0.25 μm thickness). Injection temperature was 280°C and oven ramp was: 180°C for 2 min, 6°C·min^-1^ ramp to 215°C, held for 1 min, and then 40°C·min^-1^ to 320°C, held for 22 min. The flow rate of the carrier gas, Helium, was 1.0 mL·min^-1^. Soluble sugars were identified and quantified (Chemstation Quantiation Process Program, Agilent Technologies, Santa Clara, CA) by comparison with standard sugars and the internal standard, phehyl β-D glucopyranoside (Sigma-Aldrich, St. Louis, MO).

### Statistical analysis

The data were subjected to one-way analysis of variance using Minitab statistical software (Minitab Inc., State College, PA). The model tested for main effects of different treatments. When appropriate, means were separated using LSD (α=0.05). The correlation between bud freezing tolerance and sugar concentration was determined using Pearson Correlation Analysis.

## Results

### Effect of ABA on freezing tolerance of greenhouse-grown and field-grown grapevines

**Figure 1:**
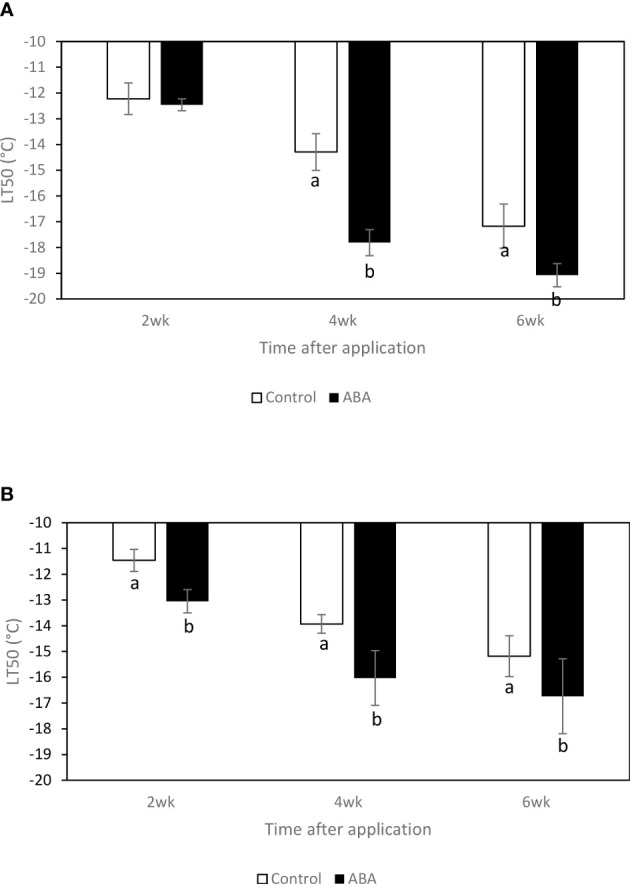
Effect of ABA on bud freezing tolerance (LT50) of greenhouse-grown grapevines, **(A)** ‘Chambourcin’ and **(B)** ‘Cabernet franc’. Bars with different letters are significantly different at *p* ≤ 0.05. (n = 4). Standard errors are presented.

In the greenhouse, between 2wk and 6wk after ABA application, the LT50s of ‘Chambourcin’ and ‘Cabernet franc’ grape buds consistently decreased by 6 and 2.5°C on average, respectively. In ‘Chambourcin’ grape buds, ABA treatment started to affect freezing tolerance 4wk after ABA application and decreased the LT50s by 3.8°C on average (**Figure 1A**). In ‘Cabernet franc’ grape buds, ABA treatment started to affect freezing tolerance 2wk after ABA application and decreased the LT50s by 2.5°C on average (**Figure 1B**). In sum, the LT50s between 2wk and 6wk decreased and ABA-increased freezing tolerance was consistent between 2wk and 6wk. The data of the freezing tolerance in the field experiments of ‘Cabernet franc’ and ‘Chambourcin’ has been reported by Zhang and Dami (2012a) and Zhang and Dami (2012b), respectively. The lowest temperatures recorded in 2011 were -23.3°C on 24 January in Kingsville and -21.3°C on January 13 in Wooster (**Supplemental Figures 1**, **2**). Overall, ABA at veraison and post-veraison increased freezing tolerance in ‘Cabernet franc’ (Zhang and Dami, 2012a) and in ‘Chambourcin (Zhang and Dami, 2012b) in midwinter.

### Effect of ABA on the seasonal changes of soluble sugar concentrations in the field-grown grapevines

In ‘Chambourcin’ grape buds, the concentrations of fructose, glucose, sucrose, galactinol consistently increased from late fall to mid-winter and did not reach their maximum values until February (**Table 1**). The concentrations of raffinose and stachyose reached peak values in December, and then started to decrease in January and February (**Table 1**). In ‘Cabernet franc’ grape buds, the concentrations of soluble sugars followed a similar pattern as in ‘Chambourcin’ by reaching maximum concentrations in January (**Table 2**). Raffinose and stachyose reached peak values in November, and then started to decrease in December and January (**Table 2**).

**Table 1:** Effect of ABA on the seasonal changes of the sugar concentrations (mg/g dry wt) in ‘Chambourcin’ buds.

Sugar	Treatment*	29 Oct.	22 Nov.	15 Dec.	15 Jan.	27 Feb.
Fructose	Control	18.3 ± 1.6	29.6 ± 2.2 c	31.1 ± 1.3 c	34.0 ± 1.6 b	37.7 ± 2.1 b
	4V	18.7 ± 0.6	33.5 ± 1.0ab	37.8 ± 2.0 b	46.2 ± 2.6 a	44.9 ± 1.9 a
	4V20	18.5 ± 0.8	34.6 ± 1.7 a	49.0 ± 0.2 a	41.9 ± 2.5 a	44.5 ± 2.7 a
	4V40	17.5 ± 1.3	35.2 ± 1.6 a	31.7 ± 1.0 bc	26.5 ± 0.6 b	34.6 ± 2.4 b
	6V	16.9 ± 1.0	35.0 ± 1.9 a	44.9 ± 1.5 a	40.0 ± 1.3 a	40.3 ± 2.3 b
	6V20	19.0 ± 1.8	31.5 ± 1.7 bc	34.4 ± 1.5 bc	47.0 ± 2.0 a	38.1 ± 2.3 b
	6V40	15.8 ± 1.2	29.6 ± 0.2 c	28.0 ± 1.7 c	42.9 ± 1.6 a	40.0 ± 2.3 b
	p-value	ns	0.001	<0.001	0.048	0.001
Glucose	Control	13.8 ± 0.8 b	22.6 ± 0.8 c	23.9 ± 1.1 b	27.9 ± 1.2 b	26.9 ± 1.4 c
	4V	14.2 ± 0.7 b	24.3 ± 1.6 b	25.4± 1.9ab	28.4 ± 1.3 b	27.9 ± 1.2 c
	4V20	14.3 ± 0.8 b	25.0 ± 0.8 ab	26.5 ± 1.2 ab	28.7 ± 1.7 b	30.0 ± 1.7 b
	4V40	13.1 ± 0.8 b	25.2 ± 0.8ab	28.3 ± 1.2 ab	28.6 ± 1.4 b	31.9± 1.6 ab
	6V	17.3 ± 1.7 a	26.5 ± 2.0 a	29.6 ± 1.8 a	28.2 ± 1.3 b	32.2 ± 1.1 ab
	6V20	17.3 ± 0.6 a	25.9 ± 1.4 a	29.1 ± 1.2 a	29.9 ± 1.3 a	33.4 ± 1.7 a
	6V40	13.6 ± 0.6 b	25.2 ± 1.2 ab	29.7 ± 1.5 a	32.1 ± 1.7 a	32.8 ± 1.8 ab
	p-value	<0.001	<0.001	0.024	0.001	0.011
Sucrose	Control	17.5 ± 0.9	16.5 ± 1.1 c	26.3 ± 1.8 d	23.6 ± 1.6 c	32.4 ± 1.4 c
	4V	19.0 ± 1.2	23.8 ± 1.9 b	33.0 ± 2.1 ab	41.6 ± 1.9 a	43.2 ± 1.6 a
	4V20	18.3 ± 1.5	23.8 ± 1.3 b	33.0 ± 2.1 ab	41.0 ± 1.0 a	41.0 ± 1.1 a
	4V40	18.5 ± 1.1	23.3 ± 1.3 b	28.5 ± 1.3 c	33.1 ± 1.3 b	36.6 ± 1.1 ab
	6V	19.0 ± 1.0	27.0 ± 1.6 a	34.5 ± 1.1 a	42.3 ± 1.3 a	35.2 ± 1.1 b
	6V20	20.0 ± 1.4	27.0 ± 0.7 a	35.5± 2.1 a	34.9 ± 1.0 b	38.2 ± 1.7 ab
	6V40	17.8 ± 1.1	22.0 ± 1.2 b	31.0 ± 2.1 b	36.0 ± 1.1 b	31.0 ± 1.8 c
	p-value	ns	<0.001	0.015	0.001	<0.001
Myo-inositol	Control	13.1 ± 0.5	21.2 ± 1.7	13.6 ± 0.7	8.6 ± 0.5	11.4 ± 0.8
	4V	14.4 ± 0.4	22.8 ± 1.0	14.4 ± 0.4	8.5 ± 0.8	11.9 ± 1.0
	4V20	13.2 ± 1.1	21.2 ± 0.5	13.3 ± 0.8	8.7 ± 0.8	10.2 ± 1.2
	4V40	14.4 ± 0.3	20.9 ± 1.1	14.0 ± 0.7	7.5 ± 1.8	13.4 ± 2.1
	6V	13.7 ± 0.5	20.2 ± 1.8	13.9 ± 0.2	9.0 ± 1.0	12.7 ± 1.2
	6V20	14.1 ± 0.4	20.1 ± 1.0	14.5 ± 0.9	10.3 ± 1.5	13.4 ± 2.0
	6V40	14.3 ± 0.6	22.5 ± 0.2	15.6 ± 1.0	12.5 ± 1.4	13.2 ± 1.4
	p-value	ns	ns	ns	ns	ns
Galactinol	Control	0.30 ± 0.04	0.42 ± 0.05 ab	0.54 ± 0.06	0.46 ± 0.03	0.83 ± 0.15 ab
	4V	0.27 ± 0.02	0.47 ± 0.03 a	0.53 ± 0.07	0.54 ± 0.04	0.83 ± 0.13 ab
	4V20	0.20 ± 0.02	0.44 ± 0.05 ab	0.51 ± 0.05	0.47 ± 0.05	0.86 ± 0.15 a
	4V40	0.30 ± 0.02	0.35 ± 0.05 b	0.45 ± 0.09	0.52 ± 0.07	0.63 ± 0.15 ab
	6V	0.31 ± 0.03	0.43 ± 0.07 ab	0.49 ± 0.03	0.54 ± 0.04	0.68 ± 0.06 ab
	6V20	0.30 ± 0.04	0.41 ± 0.07 ab	0.43 ± 0.02	0.54 ± 0.02	0.48 ± 0.03 b
	6V40	0.29 ± 0.02	0.51 ± 0.02 a	0.48 ± 0.07	0.62 ± 0.05	0.49 ± 0.02 b
	p-value	ns	0.046	Ns	ns	0.019
Raffinose	Control	2.03 ± 0.19 c	2.73 ± 0.16 c	2.94 ± 0.08 c	1.37 ± 0.17 ab	0.48 ± 0.03 ab
	4V	2.36 ± 0.07 ab	3.29± 0.14a	3.31 ± 0.11 bc	1.60 ± 0.15 a	0.44 ± 0.02 b
	4V20	2.62 ± 0.17 a	3.16 ± 0.20 ab	3.59 ± 0.19 bc	1.39 ± 0.11 ab	0.65 ± 0.02 a
	4V40	2.13 ± 0.08 bc	2.80 ± 0.11 c	3.22 ± 0.10 bc	1.54 ± 0.06 a	0.54 ± 0.03 ab
	6V	2.54 ± 0.15 a	3.18 ± 0.125 ab	4.58 ± 0.14 a	1.40 ± 0.07 ab	0.45 ± 0.02 b
	6V20	2.37 ± 0.21 ab	2.88± 0.07 bc	4.00 ± 0.15 b	1.64 ± 0.10 a	0.49 ± 0.03 ab
	6V40	2.23 ± 0.11 bc	3.19 ± 0.12 ab	3.26 ± 0.10 bc	1.60 ± 0.15 a	0.41 ± 0.02 b
	p-value	0.003	0.013	<0.001	0.002	<0.001
Stachyose	Control	1.30 ± 0.13 b	1.75 ± 0.11 b	2.21 ± 0.16 bc	2.02 ± 0.18 c	1.63 ± 0.07 ab
	4V	1.42 ± 0.09 ab	2.50 ± 0.11 a	3.14 ± 0.17 a	2.42 ± 0.09 ab	1.63 ± 0.11 ab
	4V20	1.59 ± 0.15 a	2.44 ± 0.17 ab	2.57 ± 0.07 b	2.31 ± 0.18 b	1.64 ± 0.14 ab
	4V40	1.32 ± 0.08 b	1.98 ± 0.17 b	2.43 ± 0.08 bc	2.23 ± 0.13 b	1.51 ± 0.17 b
	6V	1.40 ± 0.06 ab	2.43 ± 0.15 ab	2.65 ± 0.14 b	2.61 ± 0.04 ab	1.72 ± 0.14 a
	6V20	1.38 ± 0.09 ab	2.20 ± 0.13 ab	2.71 ± 0.17 b	2.69 ± 0.15 a	1.46 ± 0.09 b
	6V40	1.30 ± 0.11 b	1.91 ± 0.14 b	2.06 ± 0.04 c	2.30 ± 0.12 b	1.54 ± 0.04 b
	p-value	0.033	<0.001	<0.001	0.007	0.05

*4V, 4V20, 4V40 correspond to ABA application of 400 mg/L at 50% véraison stage and 20 and 40 days after 50% véraison stage, respectively. 6V, 6V20, and 6V40 correspond to ABA application of 600 at 50% véraison stage and 20 and 40 days after 50% véraison stage, respectively. (n=4). ns, not significant. Letters indicate significant differences among means.

**Table 2:** Effect of ABA on the seasonal changes of the sugar concentrations (mg/g dry wt) in ‘Cabernet franc’.

Sugar	Treatment*	11 Oct.	25 Oct.	8 Nov.	29 Nov.	21 Dec.	18 Jan.
Fructose	Control	10.2 ± 0.2 b	16.3 ± 1.6 c	22.0 ± 1.2 b	32.9 ± 1.6 b	33.8 ± 2.2 b	35.4 ± 0.4 b
	Véraison	13.7 ± 0.6 a	17.0 ± 1.0 b	27.6 ± 1.6 a	37.4 ± 1.9 a	35.8 ± 2.3 a	37.6 ± 1.4 a
	V20	14.6 ± 1.0 a	22.3 ± 0.9 a	26.1 ± 1.3 ab	38.1 ± 1.5 a	38.0 ± 1.8 a	39.5 ± 1.5 a
	V40	12.7 ± 0.8 b	21.7 ± 1.0 a	24.0 ± 0.9 ab	33.8 ± 1.6 b	35.1 ± 1.8 b	36.7 ± 1.5 ab
	V55	N/A	16.8 ± 0.7 b	23.3± 0.8 ab	34.1 ± 1.9 b	34.7 ± 1.4 b	35.6 ± 1.3 b
	p-value	0.001	<0.001	0.046	<0.001	0.011	0.038
Glucose	Control	18.9 ± 0.4 b	19.9 ± 1.6 c	22.7 ± 1.6 c	32.1 ± 1.9 c	38.6 ± 1.5 b	36.0 ± 2.0 b
	Véraison	19.9 ± 0.6 b	24.2 ± 2.0 a	26.5 ± 1.9 a	38.1 ± 1.5 a	37.5 ± 1.2 b	39.8 ± 1.8 a
	V20	20.9 ± 0.7 a	23.7 ± 1.8 a	24.3 ± 1.3 b	38.4 ± 1.5 a	43.6 ± 1.9 a	42.9 ± 0.6 a
	V40	20.0 ± 1.4 a	22.5 ± 1.6 b	24.1 ± 1.2 b	36.3 ± 1.9 b	38.7 ± 1.4 b	40.2 ± 1.2 a
	V55	N/A	22.2 ± 1.1 b	25.3 ± 0.9 a	33.3 ± 2.0 c	36.5 ± 1.2 c	37.5 ± 1.7 b
	p-value	0.049	0.046	0.022	0.01	0.038	0.035
Sucrose	Control	9.6 ± 0.5 b	15.2 ± 0.8 d	24.3 ± 0.8 b	22.9 ± 1.8 b	28.5 ± 1.7 bc	30.4 ± 0.8 b
	Veraison	13.4 ± 0.8 a	28.0 ± 0.6 b	29.5 ± 1.0 a	27.7 ± 2.2 a	31.8 ± 1.6 a	32.5 ± 1.4 a
	V20	13.0 ± 0.3 a	31.2 ± 1.8 a	32.0 ± 1.6 a	28.1 ± 1.4 a	25.9 ± 1.4 c	32.6 ± 1.2 a
	V40	9.3 ± 0.6 b	19.1 ± 1.0 c	23.2 ± 1.1 bc	21.0 ± 1.1 b	29.2 ± 1.6 ab	31.6 ± 1.5 ab
	V55	N/A	16.6 ± 1.5 cd	20.9 ± 0.8 c	22.1 ± 1.1 b	26.2 ± 1.2 bc	32.1 ± 2.2 a
	p-value	<0.001	<0.001	<0.001	<0.001	0.006	0.041
Myo-inositol	Control	3.9 ± 0.5	6.5 ± 0.3	3.3 ± 0.8	3.8 ± 0.3	2.9 ± 0.3	3.1 ± 0.4
	Veraison	4.1 ± 0.7	5.6 ± 0.1	3.6 ± 0.1	3.3 ± 0.5	2.9 ± 0.1	2.8 ± 0.3
	V20	4.8 ± 1.3	5.7 ± 0.9	3.2 ± 0.3	3.6 ± 0.5	2.9 ± 0.9	3.4 ± 0.5
	V40	4.1 ± 1.3	6.1 ± 0.4	3.8 ± 0.1	3.5 ± 0.4	3.7 ± 0.7	3.5 ± 0.4
	V55	N/A	4.4 ± 0.3	3.2 ± 0.5	2.1 ± 0.5	2.7 ± 0.8	3.4 ± 0.4
	p-value	ns	ns	ns	ns	ns	ns
Galactinol	Control	0.23 ± 0.02 b	0.39 ± 0.05 a	0.22 ± 0.07	0.26 ± 0.01	0.20 ± 0.02	0.19 ± 0.02 b
	Veraison	0.41 ± 0.12 a	0.27 ± 0.01 b	0.24 ± 0.02	0.23 ± 0.03	0.22 ± 0.01	0.19 ± 0.01 b
	V20	0.33 ± 0.12 ab	0.25 ± 0.04 b	0.20 ± 0.02	0.26 ± 0.03	0.20 ± 0.04	0.22 ± 0.02 a
	V40	0.23 ± 0.03 b	0.37 ± 0.08 a	0.25 ± 0.02	0.24 ± 0.02	0.25 ± 0.04	0.23 ± 0.01 a
	V55	N/A	0.26 ± 0.02 b	0.21 ± 0.03	0.20 ± 0.03	0.29 ± 0.02	0.24 ± 0.02 a
	p-value	0.046	0.005	ns	ns	ns	0.008
Raffinose	Control	0.25 ± 0.02	0.55 ± 0.06 bc	0.82 ± 0.08 c	0.67 ± 0.05 c	0.67 ± 0.04 c	0.26 ± 0.04 c
	Veraison	0.37 ± 0.03	0.60 ± 0.02 b	0.99 ± 0.09 bc	0.85 ± 0.05 ab	1.01 ± 0.09 a	0.42 ± 0.04 a
	V20	0.34 ± 0.02	0.72 ± 0.06 a	1.34 ± 0.04 a	0.89 ± 0.04 a	1.04 ± 0.05 a	0.38 ± 0.02 a
	V40	0.33 ± 0.02	0.78 ± 0.09 a	1.38 ± 0.02 a	0.68 ± 0.05 bc	0.82 ± 0.08 b	0.32 ± 0.01 b
	V55	N/A	0.47 ± 0.08 c	1.09 ± 0.07 b	0.47 ± 0.12 ab	0.87 ± 0.14 bc	0.30 ± 0.02 bc
	p-value	ns	<0.001	<0.001	<0.001	0.008	<0.001
Stachyose	Control	0.66 ± 0.08 c	0.31 ± 0.03 c	0.30 ± 0.08 b	0.37 ± 0.02 b	0.28 ± 0.04 c	0.25 ± 0.03 b
	Veraison	1.00 ± 0.11 a	0.43 ± 0.01 bc	0.36 ± 0.05 a	0.40 ± 0.06 a	0.37 ± 0.02 a	0.29 ± 0.02 a
	V20	0.95 ± 0.04 ab	0.55 ± 0.12 ab	0.39 ± 0.04 a	0.38 ± 0.11 a	0.36 ± 0.02 a	0.30 ± 0.03 a
	V40	0.83 ± 0.10 b	0.67 ± 0.15 a	0.36 ± 0.02 a	0.34 ± 0.04 b	0.34 ± 0.03 ab	0.30 ± 0.01 a
	V55	N/A	0.45 ± 0.08 bc	0.31 ± 0.10 b	0.27 ± 0.03 b	0.29 ± 0.06 bc	0.32 ± 0.02 a
	p-value	0.002	0.026	0.05	0.042	0.033	0.027

Veraison, V20, V40 and V55, correspond to ABA application of 600 mg/L at 50% véraison stage and 20 days after 50% véraison stage, respectively. 6V and 6V20 correspond to ABA application of 600 at 50% véraison stage and 20 days after 50% véraison stage, respectively. ns, not significant; N/A, not available. Letters indicate significant differences among means.

Compared to control, some ABA treatments increased the concentrations of fructose, glucose, sucrose, galactinol, raffinose, and stachyose during the dormant season in both cultivars. In ‘Chambourcin’ buds, ABA increased the concentrations of the above sugars, relative to the control, on average by 12% (galactinol) to 58% (fructose) (**Table 1**). In ‘Cabernet franc’ buds, ABA increased the concentrations of the above sugars on average by 6% (glucose) to 100% (galactinol) (**Table 2**).

### Effect of ABA on the sugar concentrations of greenhouse-grown grape leaves and buds

**Figure 2:**
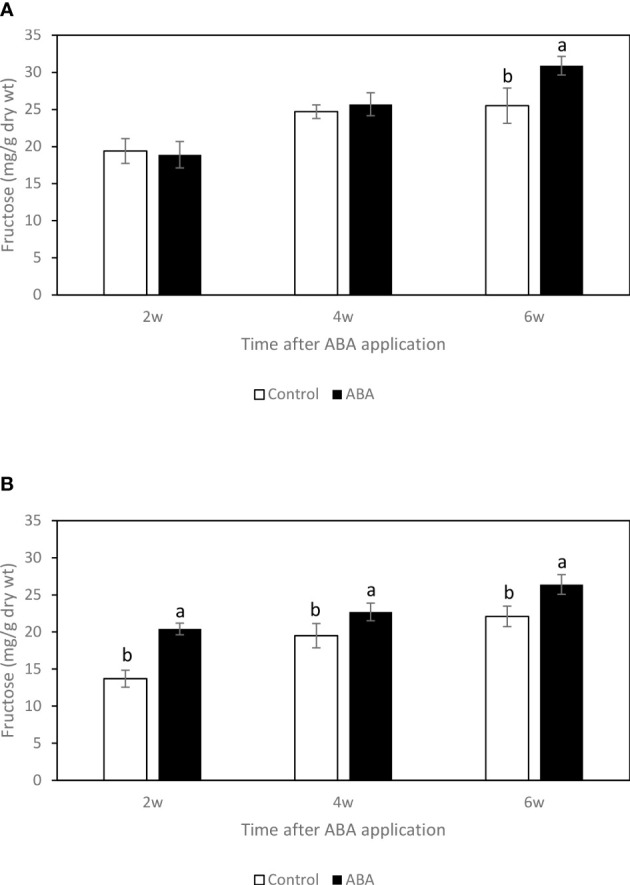
Effect of ABA on the bud concentrations of fructose in greenhouse-grown grapevines **(A)**’Chambourcin’ and **(B)** ‘Cabernet franc’. Bars with different letters are significantly different at *p* ≤ 0.05. (n = 4). Standard errors are presented.

**Figure 3:**
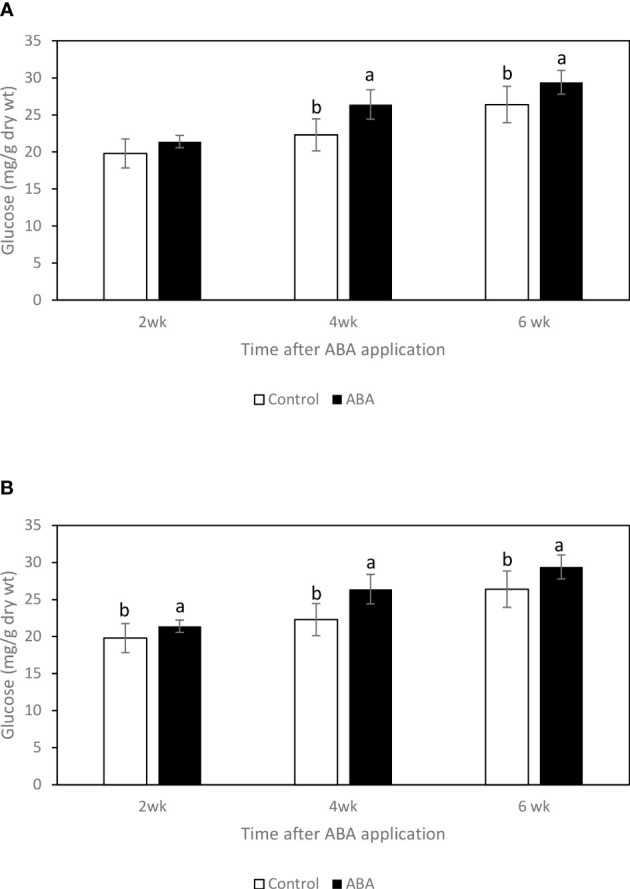
Effect of ABA on the bud concentrations of glucose in greenhouse-grown grapevines **(A)** ‘Chambourcin’ and **(B)** ‘Cabernet franc’. Bars with different letters are significantly different at *p* ≤ 0.05. (n = 4). Standard errors are presented.

**Figure 4:**
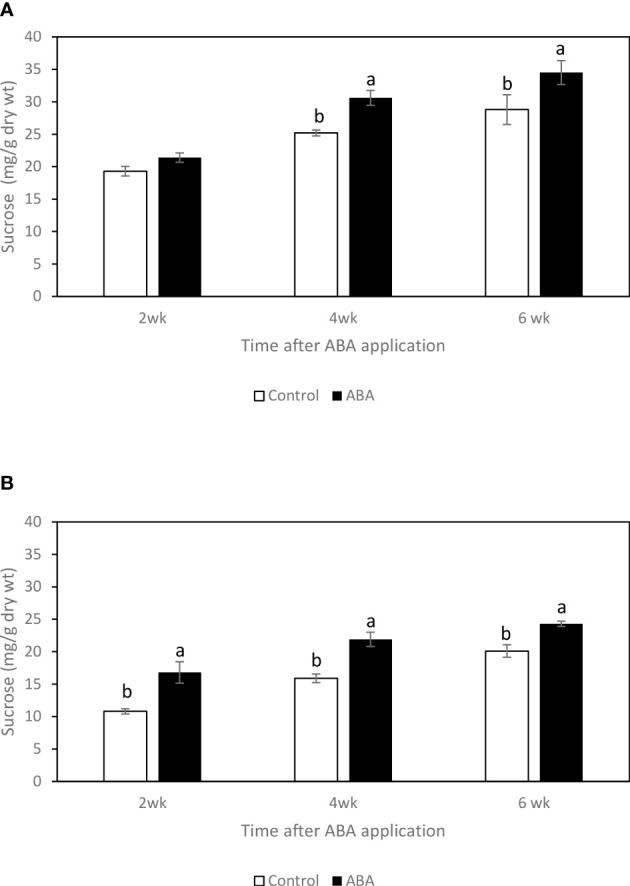
Effect of ABA on the bud concentrations of sucrose in greenhouse-grown grapevines **(A)** ‘Chambourcin’ and **(B)** ‘Cabernet franc’. Bars with different letters are significantly different at *p* ≤ 0.05. (n = 4). Standard errors are presented.

**Figure 5:**
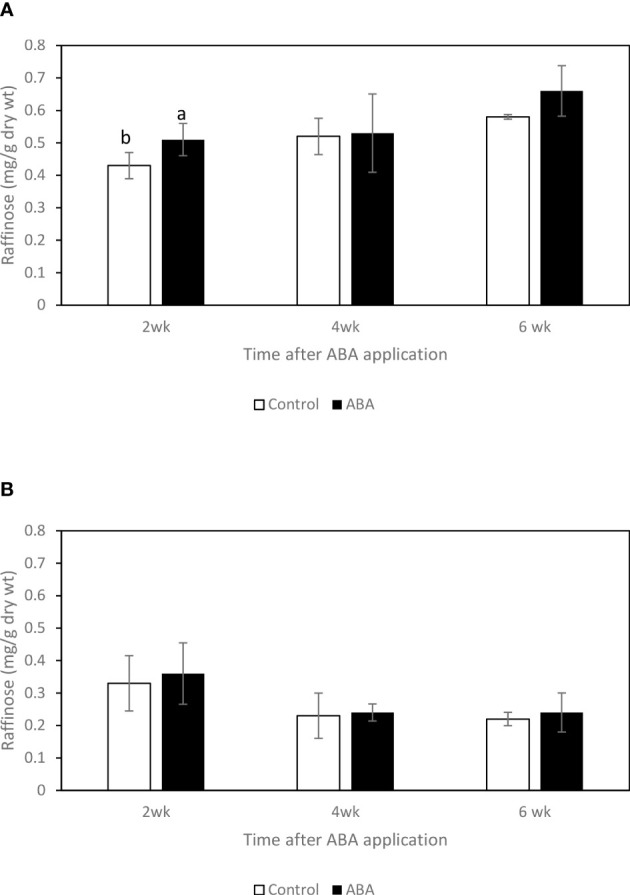
Effect of ABA on the bud concentrations of raffinose in greenhouse-grown grapevines **(A)** ‘Chambourcin’ and **(B)** ‘Cabernet franc’. Bars with different letters are significantly different at *p* ≤ 0.05. (n = 4). Standard errors are presented.

Between 2wk and 6wk after ABA application, there were no trends of soluble sugar concentrations in either ‘Chambourcin’ or ‘Cabernet franc’ grape leaves (data not shown). In ‘Chambourcin’ buds, the concentrations of fructose, glucose, and sucrose increased on average by 47%, 35%, and 56%, respectively (**Figures 2A**, **3A**, **4A**). In ‘Cabernet franc’ buds, the concentrations of fructose, glucose, and sucrose increased on average by 42%, 37%, and 82%, respectively (**Figures 2B**, **3B**, **4B**). The ABA treatment significantly increased the concentrations of the above three soluble sugars. In ‘Chambourcin’ buds, ABA treatment increased the concentrations of fructose, glucose, and sucrose on average by 8%, 13%, and 17%, respectively (**Figures 2A**, **3A**, **4A**). In ‘Cabernet franc’ buds, ABA treatment increased the concentrations of fructose, glucose, and sucrose on average by 26%, 22%, and 27%, respectively (**Figures 2B**, **3B**, **4B**). There were variations of galactinol (data not shown) and raffinose (**Figure 5**) concentrations between 2wk and 6wk after ABA application. However, neither the trend of variation nor ABA effect was significant. The only significant difference was at 2wk in ‘Chambourcin’ with higher raffinose in ABA-treated than in control (**Figure 5**).

### Association between freezing tolerance and soluble sugar concentrations of grape buds

**Figure 6:**
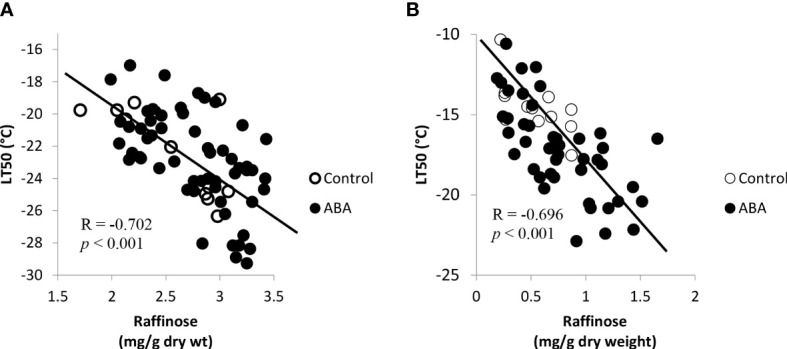
Correlations between freezing tolerance (LT50) and the bud raffinose concentrations in field grown grapevines during the acclimation stage, October – December for **(A)** ‘Chambourcin’ and October – November for **(B)** ‘Cabernet franc’. (n = 4).

The concentrations of fructose, glucose, and sucrose consistently correlated with freezing tolerance of grape buds in the greenhouse and field (**Table 3**). The concentrations of these sugars increased while the LT50 decreased (freezing tolerance increased). The correlation between galactinol and freezing tolerance varied between greenhouse and field study and between ‘Chambourcin’ and ‘Cabernet franc’. In the field study, the correlation between raffinose and freezing tolerance was not significant for either cultivar. The raffinose concentration did not correlate with bud freezing tolerance because it increased in late fall and reached the peak in early winter. However, during the early acclimation stage (October – December for ‘Chambourcin’ grapevines and October – November for ‘Cabernet franc’ grapevines), the raffinose concentrations negatively correlated with freezing tolerance expressed as LT50s in ‘Chambourcin’ (R=-0.702, *p*<0.001, **Figure 6A**) and ‘Cabernet franc’ (R=-0.696, *p*<0.001, **Figure 6B**). There was also correlation between the other sugars and freezing tolerance during the early acclimation stage (**Supplemental Table 1**). In the greenhouse study, the correlation was significant for ‘Chambouricn’ only (**Table 3**).

**Table 3:** Correlations between soluble sugar concentrations and freezing tolerance (LT50) of grape buds.

Sugars	Field		Greenhouse	
	Chambourcin	Cabernet franc	Chambourcin	Cabernet franc
Fructose	-0.593***^z^	-0.861***	-0.822***	-0.756***
Glucose	-0.708***	-0.790***	-0.833***	-0.666***
Sucrose	-0.719***	-0.769***	-0.928***	-0.763***
Galactinol	-0.411*	0.314**	0.442*	-0.130 ns
Raffinose	0.117 ns	0.049 ns	0.559**	0.284 ns

^z^ ns, *, **, and *** No significant, significant at p ≤ 0.05, 0.01, and 0.001, respectively.

## Discussion

### Effect of ABA on freezing tolerance of grape bud under greenhouse conditions

ABA treatment increased freezing tolerance of both ‘Chambourcin’ and ‘Cabernet franc’ grapevines under greenhouse conditions. ABA treatment started to increase freezing tolerance 2wk after application. In the greenhouse experiment, the ABA-treated grapevines had higher freezing tolerance than untreated ones. In ‘Chambourcin’ buds, the effect was seen 4wk after application with a rapid decrease of LT50 between 2wk and 4wk after ABA application. The greenhouse study confirmed other findings that ABA treatment increased freezing tolerance of greenhouse-grown ‘Cabernet franc’ (Wang et al., 2020), and field-grown ‘Cabernet franc’ (Zhang and Dami, 2012a), ‘Pinot gris’ (Li and Dami, 2016), and ‘Chambourcin’ grapevines (Zhang and Dami, 2012b). It is also suggested that it takes time (2wk to 4wk) for grapevines to show the effect of ABA on freezing tolerance.

### Seasonal changes of soluble sugar concentrations and their correlations with freezing tolerance of grape buds

Soluble sugars have been suggested to play an important role in protecting cells from cold damage. In the greenhouse and field studies, three soluble sugars, fructose, glucose, and sucrose, consistently increased during the winter season and reached their maximum values when the grape buds were at their maximum freezing tolerance. This result is consistent with previous studies on the seasonal carbohydrate changes in grape buds (Hamman et al., 1996; Jones et al., 1999). Soluble sugar concentrations increase in the fall in response to low temperatures, reach a maximum during the coldest months in mid-winter, and decrease in the spring (Sakai and Larcher, 1987). The raffinose family oligosaccharides (RFO) appear to be the most important in mediating FT, as they change exclusively with cold acclimation. Even though raffinose is a very minor carbohydrate in grape tissues there is reason to believe that it is very important in freezing tolerance of *Vitis* species. In fact, raffinose has been shown to play a cryoprotective role by protecting cell membranes, stabilizing proteins, and retaining enzyme activities during a freeze-thaw event (Anchordoguy et al., 1987; Hincha et al., 1993; Stushnoff et al., 1997). However, the relationship between raffinose and LT50 was not consistent in grapes. For example, some reports demonstrated that the maximum level of freezing tolerance of grape buds was not always associated with the highest level of raffinose concentration in those tissues (Koussa et al., 1998; Jones et al., 1999). In Arabidopsis, it has been suggested that raffinose is not essential for freezing tolerance development. The raffinose-deficient mutant could still develop cold acclimation without raffinose accumulation within the tissues (Zuther et al., 2004). In this study, we found a similar discrepancy where raffinose concentration in buds peaked in late fall to early winter (October-December) and did not correspond to the maximum level of freezing tolerance which was reached in January. These results also corroborate a French report that showed that raffinose peaked in November on Chardonnay vines grown in the Burgundy region (Koussa et al., 1998). The early findings coupled with the studies described above may explain that while the correlation between LT50 and raffinose exists, it does not support its critical role to maximize freezing tolerance in mid-winter like in other plant species. For these reasons, a different mechanism to explain the role of raffinose in grapes is proposed as follows. First, raffinose accumulation has been demonstrated as an early response triggered by low but non-freezing temperatures that prepare buds for dormancy and cold acclimation (Grant et al., 2009). Second, in the field study, bud tissue dehydration began early in the fall (Zhang and Dami, 2012a; Zhang and Dami, 2012b) coinciding with raffinose accumulation which peaked before the occurrence of sub-freezing temperatures. In the greenhouse study, raffinose concentration also peaked before the occurrence of increased freezing tolerance. Therefore, it is suggested that raffinose might play a more important role in desiccation rather than freezing tolerance in grapevines. However, this needs further investigation.

Raffinose has been demonstrated to stabilize membrane during desiccation by forming hydrogen bonds and substituting for water during desiccation (Crowe et al., 1989; Hoekstra et al., 2001). Furthermore, raffinose has been suggested as an osmoprotectant like proline (Gilmour et al., 2000; Taji et al., 2002). In the greenhouse study, at 2wk after ABA application, it was observed that the sucrose concentration significantly increased, the LT50s started to decrease, and the raffinose decreased. It is suggested that when grapevines start to increase freezing tolerance by accumulating soluble sugars, raffinose is one source for that. The field study showed that raffinose concentration was at the lowest level in mid-winter, which is likely metabolized into small sugars such as fructose and glucose which are important for freeze protection in mid-winter. Actually, this has been verified in previous studies which reported that glucose and fructose, but not raffinose are the predominant sugars during maximum hardiness in the ‘Riesling’ and ‘Chardonnay’ (*Vitis vinifera*) (Hamman et al., 1996; Koussa et al., 1998; Jones et al., 1999). These small sugars protect against freezing by depression of the nucleating temperature to promote supercooling (Sakai and Larcher, 1987; Crowe et al., 1989; Takata et al., 2007). This hypothesis, however, needs further investigation.

### Effect of ABA on the soluble sugar accumulation

Both in the greenhouse- and field-grown grape buds, it has been observed that fructose, glucose, and sucrose increased in ABA-treated grape buds. Additionally, ABA promoted the accumulation of raffinose in buds from field and greenhouse-grown ‘Cabernet franc’ grapevines (2wk after application). The effect of ABA on the productions of these sugars has also been found in other plants. It has been reported that ABA treatment significantly promoted the accumulation of fructose, glucose, sucrose, and raffinose in the seedling of cucumber (*Cucumis sativus* L.) (Meng et al., 2008). In winter rapeseed (*Brassica napus* L.) shoots, exogenous ABA treatment significantly promoted the accumulation of soluble sugars with the increased freezing tolerance (Burbulis et al., 2010). In barley (Hordeum vulgare *L.*), exogenous ABA application increased freezing tolerance of plant tissues by promoting the production of sucrose (Bravo et al., 1998). Raffinose is closely related to the ABA-inducible desiccation tolerance, especially in seeds. For instance, it has been reported that exogenous ABA treatment can significantly increase the raffinose concentration in alfalfa (*Medicago sativa* L.) seeds by increasing the galactinol synthase activity (Blochl et al., 2005).

## Conclusion

In summary, this study has demonstrated that fructose, glucose, and sucrose are the main soluble sugars that correlate with freezing tolerance of grape buds. Exogenous ABA application increased freezing tolerance of grape buds by promoting the accumulation of these soluble sugars. This study also suggested that ABA application can promote raffinose accumulation, but this sugar may play an important role in the early acclimation stage for increasing the desiccation tolerance. The preliminary result suggested that chronologically raffinose accumulates first in the buds before cold treatment. Then, a decrease of raffinose concentration coincided with the increase of smaller sugars, sucrose, fructose, and glucose. The accumulations of the latter sugars correspond to the maximum increase of freezing tolerance.

Cold damaging events are predicted to be exacerbated with the warming trend of climate (Schultze and Sabbatini, 2019). In this era of climate change and increasingly variable weather, there is a great need to advance the science of freezing tolerance in plants by developing more resilient grapevines to cold damage. This study demonstrates the importance of soluble sugars, including RFO in gaining freezing tolerance and that ABA can be used as a cultural practice to enhance freezing tolerance in grapevines and reduce economic losses associated with cold damage.

## Data availability statement

The original contributions presented in the study are included in the article/**Supplementary Material**. Further inquiries can be directed to the corresponding author.

## Author contributions

ID designed experiments and developed treatments and data collection and co-wrote manuscript. YZ conducted experiments, collected data, analyzed results and co-wrote manuscript. All authors contributed to the article and approved the submitted version.

## References

[B1] Allison S. Chang B. Randolph T. Carpenter J. (1999). Hydrogen bonding between sugar and protein is responsible for inhibition of dehydration-induced protein unfolding. Arch. Biochem. Biophys. 365, 289–298. doi: 10.1006/abbi.1999.1175 10328824

[B2] Anchordoguy T. Rudolph A. Carpenter J. Crowe J. (1987). Modes of interaction of cryoprotectants with membrane phospholipids during freezing. Cryobiology 24, 324–331. doi: 10.1016/0011-2240(87)90036-8 3621976

[B3] Bhyan S. B. Minami A. Kaneko Y. Suzuki S. Arakawa K. Sakata Y.. (2012). Cold acclimation in the moss physcomitrella patens involves abscisic acid-dependent signaling. J. Plant Physiol. 169, 137–145. doi: 10.1016/j.jplph.2011.08.004 21958596

[B4] Blochl A. Grenier-de March G. Sourdioux M. Peterbauer T. Richter A. (2005). Induction of raffinose oligosaccharide biosynthesis by abscisic acid in somatic embryos of alfalfa (Medicago sativa l.). Plant Sci. 168, 1075–1082. doi: 10.1016/j.plantsci.2004.12.004

[B5] Bravo L. A. Zuniga G. E. Alberdi M. Corcuera L. J. (1998). The role of ABA in freezing tolerance and cold acclimation in barley. Physiol. Plant 103, 17–23. doi: 10.1034/j.1399-3054.1998.1030103.x

[B6] Burbulis N. Jonytiene V. Kupriene R. Blinstrubiene A. Liakas V. (2010). Effect of abscisic acid on cold tolerance in brassica napus shoots cultured *in vitro* . J. Food Agric. Environ. 8, 698–701.

[B7] Chai F. Liu W. Xiang Y. Meng X. Sun X. Cheng C.. (2019). Comparative metabolic profiling of vitis amurensis and vitis vinifera during cold acclimation. Horticult. Res. 6 (1), 1–12. doi: 10.1038/s41438-018-0083-5 PMC6312538 30603094

[B8] Chao W. S. Foley M. E. Horvath D. P. Anderson J. V. (2007). International journal of plant developmental biology ©2007 global science books signals regulating dormancy in vegetative buds. International Journal of Plant Developmental Biology ©2007 Global Science Books. 49–56.

[B9] Eichhorn K. W. Lorenz D. H. (1977). Phenological development stages of the grapevine. Nachrichtenblatt Des Deutschen Pflanzenschutzdienstes 29, 119–120.

[B10] Crowe J. Hoekstra F. Crowe L. (1989). Membrane phase transitions are responsible for imbibitional damage in dry pollen. Proc. Natl. Acad. Sci. U. S. A. 86, 520–523. doi: 10.1073/pnas.86.2.520 16594011 PMC286503

[B11] Gilmour S. Sebolt A. Salazar M. Everard J. Thomashow M. (2000). Overexpression of the arabidopsis CBF3 transcriptional activator mimics multiple biochemical changes associated with cold acclimation. Plant Physiol. 124, 1854–1865. doi: 10.1104/pp.124.4.1854 11115899 PMC59880

[B12] Grant T. N. Dami I. E. Ji T. Scurlock D. Streeter J. (2009). Variation in leaf and bud soluble sugar concentration among vitis genotypes grown under two temperature regimes. Can. J. Plant Sci. 89, 961–968. doi: 10.4141/CJPS08188

[B13] Hamman R. A. Dami I. E. Walsh T. M. Stushnoff C. (1996). Seasonal carbohydrate changes and cold hardiness of Chardonnay and Riesling grapevines. Am. J. Enol. Vitic. 47, 31–36. doi: 10.5344/ajev.1996.47.1.31

[B14] Hincha D. Bakaltcheva I. Schmitt J. (1993). Galactose specific lectins protect isolated thylakoids against freeze-thaw damage. Plant Physiol. 103, 59–65. doi: 10.1104/pp.103.1.59 12231914 PMC158946

[B15] Hoekstra F. Golovina E. Tetteroo F. Wolkers W. (2001). Induction of desiccation tolerance in plant somatic embryos: How exclusive is the protective role of sugars? Cryobiology 43, 140–150. doi: 10.1006/cryo.2001.2358 11846469

[B16] Jones K. Paroschy J. McKersie B. Bowley S. (1999). Carbohydrate composition and freezing tolerance of canes and buds in vitis vinifera. J. Plant Physiol. 155, 101–106. doi: 10.1016/S0176-1617(99)80146-1

[B17] Kerepesi I. Banyai-Stefanovits E. Galiba G. (2004). Cold acclimation and abscisic acid induced alterations in carbohydrate content in calli of wheat genotypes differing in frost tolerance. J. Plant Physiol. 161, 131–133. doi: 10.1078/0176-1617-00766 15002675

[B18] Koussa T. Cherrad M. Bertrand A. Broquedis M. (1998). Comparison of the contents of starch, soluble carbohydrates and abscisic acid of latent buds and internodes during the vegetative cycle of grapevine. Vitis 37, 5–10.

[B19] Kovaleski A. P. Reisch B. I. Londo J. P. (2018). Deacclimation kinetics as a quantitative phenotype for delineating the dormancy transition and thermal efficiency for budbreak in vitis species. AoB Plants 10 (5), 1–12. doi: 10.1093/AOBPLA/PLY066 PMC6207836 31572566

[B20] Li S. Dami I. E. (2016). Responses of vitis vinifera ‘Pinot gris’ grapevines to exogenous abscisic acid (ABA): I. yield, fruit quality, dormancy, and freezing tolerance. J. Plant Growth Regul. 35 (1). doi: 10.1007/s00344-015-9529-2

[B21] Londo J. P. Kovaleski A. P. Lillis J. A. (2018). Divergence in the transcriptional landscape between low temperature and freeze shock in cultivated grapevine (*Vitis vinifera* ). Horticult. Res. 5(1), 1–14. doi: 10.1038/s41438-018-0020-7 PMC5830407 29507734

[B22] Meng F. Hu L. Wang S. Sui X. Wei L. Wei Y.. (2008). Effects of exogenous abscisic acid (ABA) on cucumber seedling leaf carbohydrate metabolism under low temperature. Plant Growth Regul. 56, 233–244. doi: 10.1007/s10725-008-9303-6

[B23] Ogawa A. Yamauchi A. (2006). Root osmotic adjustment under osmotic stress in maize seedlings 2: Mode of accumulation of several solutes for osmotic adjustment in the root. Plant Prod. Sci. 9, 39–46. doi: 10.1626/pps.9.39

[B24] Sacha G. A. Nail S. L. (2009). Thermal analysis of frozen solutions: multiple glass transitions in amorphous systems. J. Pharm. Sci. 98, 3397–3405. doi: 10.1002/jps.21737 19384925

[B25] Sakai A. Larcher W. (1987). Frost survival of plants: Responses and adaptation to freezing stress Vol. 62 (Berlin Heidelberg: Springer-Verlag).

[B26] Sauter J. Wisniewski M. Witt W. (1996). Interrelationships between ultrastructure, sugar levels, and frost hardiness of ray parenchyma cells during frost acclimation and deacclimation in poplar (Populus x canadensis moench ) wood. J. Plant Physiol. 149, 451–461. doi: 10.1016/S0176-1617(96)80148-9

[B27] Schultze S. R. Sabbatini P. (2019). Implications of a climate-changed atmosphere on cool-climate viticulture. J. Appl. Meteorol. Climatol. 58 (5), 1141–1153. doi: 10.1175/JAMC-D-18-0183.1

[B28] Snyder R. L. de Melo-Abreu J. P. (2005). Frost protection: fundamentals, practice, and economics. Food and Agriculture Organization of the United Nations Rome. 2, 72.

[B29] Stushnoff C. Seufferheld M. Creegan T. (1997). Oligosaccharides as endogenous cryoprotectants in woody plants (New York, N.Y: Plenum Press Div Plenum Publishing Corp).

[B30] Suzuki M. Ishikawa M. Okuda H. Noda K. Kishimoto T. Nakamura T.. (2006). Physiological changes in gentian axillary buds during two-step preculturing with sucrose that conferred high levels of tolerance to desiccation and cryopreservation. Ann. Bot. 97, 1073–1081. doi: 10.1093/aob/mcl054 16565150 PMC2803387

[B31] Taji T. Ohsumi C. Iuchi S. Seki M. Kasuga M. Kobayashi M.. (2002). Important roles of drought- and cold-inducible genes for galactinol synthase in stress tolerance in arabidopsis thaliana. Plant J. 29, 417–426. doi: 10.1046/j.0960-7412.2001.01227.x 11846875

[B32] Takata N. Kasuga J. Takezawa D. Arakawa K. Fujikawa S. (2007). Gene expression associated with increased supercooling capability in xylem parenchyma cells of larch (Larix kaempferi). J. Exp. Bot. 58, 3731–3742. doi: 10.1093/jxb/erm223 18057043

[B33] Wang H. Blakeslee J. J. Jones M. L. Chapin L. J. Dami I. E. (2020). Exogenous abscisic acid enhances physiological, metabolic, and transcriptional cold acclimation responses in greenhouse-grown grapevines. Plant Sci. 1–13. doi: 10.1016/j.plantsci.2020.110437 32081274

[B34] Wang S. Hu L. Sun J. Sui X. Wei Y. Zhang Z. (2012). Effects of exogenous abscisic acid on leaf carbohydrate metabolism during cucumber seedling dehydration. Plant Growth Regul. 66, 87–93. doi: 10.1007/s10725-011-9632-8

[B35] Wang Y. Xin H. Fan P. Zhang J. Liu Y. Dong Y.. (2021). The genome of shanputao (Vitis amurensis) provides a new insight into cold tolerance of grapevine. Plant J. 105 (6), 1495–1506. doi: 10.1111/tpj.15127 33300184

[B36] Wanner L. Junttila O. (1999). Cold-induced freezing tolerance in arabidopsis. Plant Physiol. 120, 391–399. doi: 10.1104/pp.120.2.391 10364390 PMC59277

[B37] Wolf, T. K. Pool R. M. (1987). Factors Affecting Exotherm Detection in the Differential Thermal-Analysis of Grapevine Dormant Buds. J. Am. Soc. Horticultural Sci. (0003-1062), 112 (3), 520.

[B38] Wolf T. K. Cook M. K. (1992). Seasonal deacclimation patterns of three grape cultivars at constant, warm temperature. Am. J. Enol. Viticult. 43 (2), 171–179. doi: 10.5344/ajev.1992.43.2.171

[B39] Xu R. Niimi Y. Han D. (2006). Changes in endogenous abscisic acid and soluble sugars levels during dormancy-release in bulbs of lilium rubellum. Sci. Hortic. 111, 68–72. doi: 10.1016/j.scienta.2006.08.004

[B40] Xue-Xuan X. Hong-Bo S. Yuan-Yuan M. Gang X. Jun-Na S. Dong-Gang G.. (2010). Biotechnological implications from abscisic acid (ABA) roles in cold stress and leaf senescence as an important signal for improving plant sustainable survival under abiotic-stressed conditions. Crit. Rev. Biotechnol. 30, 222–230. doi: 10.3109/07388551.2010.487186 20572794

[B41] Zhang Y. Dami I. (2012a). Folia application of abscisic acid increased freezing tolerance of field-grown vitis vinefera ‘Cabernet franc’ grapevines. Am. J. Enol. Vitic. 63, 85–93. doi: 10.5344/ajev.2012.12006

[B42] Zhang Y. Dami I. (2012b). Improving freezing tolerance of ‘Chambourcin’ grapevines with exogenous abscisic acid. HortScience 47, 1750–1757. doi: 10.21273/HORTSCI.47.12.1750

[B43] Zhang Y. Mechlin T. Dami I. (2011). Foliar application of abscisic acid induces dormancy responses in greenhouse-grown grapevines. HortScience 46, 1271–1277. doi: 10.21273/HORTSCI.46.9.1271

[B44] Zuther E. Buchel K. Hundertmark M. Stitt M. Hincha D. Heyer A. (2004). The role of raffinose in the cold acclimation response of arabidopsis thaliana. FEBS Lett. 576, 169–173. doi: 10.1016/j.febslet.2004.09.006 15474032

