# Supplementary material for: Variations of freezing tolerance and sugar concentrations of grape buds in response to foliar application of abscisic acid
Source: Front Plant Sci. 2023 Feb 17;14:1084590. doi: 10.3389/fpls.2023.1084590 (PMC9981962; doi:10.3389/fpls.2023.1084590)
Supplement: Supplementary file 1 [file DataSheet_1.docx]

**Supplemental Tables**

**Table 1.** Correlations between soluble sugar concentrations and freezing tolerance (LT50) of grape buds during the acclimation stage, October-December, for field grown ‘Chambourcin’ and October-November for field grown ‘Cabernet franc’.

| Sugars | Field | |
| --- | --- | --- |
|  | Chambourcin | Cabernet franc |
| Fructose | -0.6794***^z^ | -0.8681*** |
| Glucose | -0.7602*** | -0.7903*** |
| Sucrose | -0.7915*** | -0.7196*** |
| Myo-inositol | 0.0427 ns | - 0.3066** |
| Galactinol | -0.5455*** | 0.2743* |

^z^ ns, *, **, and *** No significant, significant at *p* ≤ 0.05, 0.01, and 0.001, respectively.

**Supplemental Figures**

**Figure 1**. Daily minimum and maximum temperatures recorded during the 2010-2011 dormant season at the Wooster location.

**Figure 2.** Daily minimum and maximum temperatures recorded during the 2010-2011 dormant season at the Kingsville location.
